# Impact of Long-Term Steroid Use on the Disposition of Patients Undergoing Transcatheter Aortic Valve Replacement: A Retrospective Nationwide Sample Analysis

**DOI:** 10.7759/cureus.38048

**Published:** 2023-04-24

**Authors:** Endurance O Evbayekha, Gabriel Alugba, Theresa O Akewe, Oyindamola O Obadare, Vanessa O Agberien, Adebola E Omogunwa, Anthony Willie, Jane N Nwafor, Adetoro T Okafor, Okelue E Okobi

**Affiliations:** 1 Internal Medicine, St. Luke's Hospital, Chesterfield, USA; 2 Internal Medicine, Delta State University, Abraka, NGA; 3 Family Medicine, University of Benin, Benin City, NGA; 4 Family and Community Medicine, Milk River Community Health Center, Milk River, CAN; 5 Internal Medicine, All saint University School of Medicine, Roseau, DMA; 6 Clinical Sciences, All Saint University School of Medicine, Roseau, DMA; 7 Population Health, Sam Houston State University, Houston, USA; 8 Emergency Medicine, Igbinedion University, Okada, NGA; 9 Internal Medicine, The University of District of Columbia, Silverspring, USA; 10 Epidemiology and Public Health, University of Minnesota School of Public Health, Minneapolis, USA; 11 Family Medicine, Medficient Health Systems, Laurel, USA; 12 Family Medicine, Lakeside Medical Center, Belle Glade, USA

**Keywords:** hospitalization, nis, transcatheter aortic valve repair, tavr, long-term steroid

## Abstract

Background

Chronic steroid use is debilitating to health, but, in some cases, it is necessary. We examined the effect of chronic steroid use on the discharge disposition of people undergoing transcatheter aortic valve replacement (TAVR).

Methods

We queried the National Inpatient Sample Database (NIS) from 2016 to 2019. We identified patients with current chronic steroid use with the International Classification of Diseases for the Tenth (ICD-10) code Z7952. Furthermore, we used the ICD-10 procedure codes for TAVR 02RF3. Outcomes were the length of hospitalization (LOS), Charlson Comorbidity Index (CCI), disposition, in-hospital mortality, and total hospital charges (THC).

Results

Between 2016 and 2019, we identified 44,200 TAVR hospitalizations, and 382,497 were on current long-term steroid therapy. Of these, 934 had current chronic steroid use and underwent TAVR (STEROID) with a mean age of 78 (SD=8.4). About 50% were female, 89% were Whites, 3.7% were Blacks, 4.2% were Hispanics, and 1.3% were Asians. Disposition was ‘home,’ ‘home with home health’ (HWHH), ‘skilled nursing home’ (SNF), ‘short-term inpatient therapy’ (SIT), ‘discharged against medical advice’ (AMA), and ‘died.’

A total of 602 (65.5%) were discharged home, 206 ( 22%) were discharged to HWHH, 109 (11.7%) to SNF, and 12 (1.28%) died. In the SIT and AMA groups, there were only three and two patients, respectively, p=0.23. The group that underwent TAVR and was not on chronic steroid therapy (NOSTEROID) had a mean age of 79 (SD=8.5), with 28731 (66.4%) being discharged home, 8399 (19.4%) to HWHH, 5319 (12.3%) to SNF, and 617 (1.43%) died p=0.17.

Comparing the STEROID vs. NONSTEROID group, according to the CCI, the STEROID group scored higher than the NOSTEROID group; 3.5 (SD=2) vs. 3 (SD=2) p=0.0001, while for LOS, it was 3.7 days (SD=4.3) vs. 4.1 days (SD=5.3), p=0.28, and the THC was $203,213 (SD=$110,476) vs. $215,858 (SD=$138,540), p=0.15.

Conclusion

The comorbidity burden of individuals on long-term steroids undergoing TAVR was slightly higher than those not on steroids undergoing TAVR. Despite this, there was no statistically significant difference in their hospital outcomes following TAVR with respect to dispositions.

## Introduction

Transcatheter aortic valve implantation (TAVR) is a feasible alternative to surgical aortic valve replacement (SAVR) in patients with symptomatic aortic stenosis [1]. Transcatheter aortic valve implantation (TAVR) has been used since 2002 [2]. In accordance with a study conducted by Overman et al., the prevalence of corticosteroid use in the United States is estimated to be 1.2% with prolonged use, and it is one of the most commonly prescribed medications worldwide [1,3]. Following surgery, the patient faces an increased risk of infection, vascular fragility, and wound healing delays, which may increase the probability of complications and harm procedural outcome measures [4]. Steroids are used as immunosuppressive therapy and an analgesic in preoperative and postoperative periods. According to Kaihara et al., immunosuppressants were not associated with increased vascular access complications and mid-term major adverse cardiac and cerebrovascular events in patients with aortic stenosis who underwent TAVR. This might imply that it may be assessed based on the underlying cause of the need for TAVR. Although several previous studies observed a correlation between chronic steroid use and periprocedural vascular complications, a well-known complication is following procedures such as TAVR [4-6]. Another study indicated no association. It seems to depend on the underlying cause for needing TAVR [7].

Steroids penetrate cell membranes and bind to hormone-receptor complexes in cell nuclei, altering ribonucleic acid (RNA) and protein synthesis, affecting the metabolism of most tissues, and leading to metabolic alterations [8]. In a previous cohort study, 12,883 participants (114 on long-term steroids) had coronary angioplasty and were evaluated for the possible risks of long-term steroid use [9]. Major vascular complications were three times more likely in the patient who used steroids (p< 0.01), and coronary perforation was three to a four-fold (p< 0.026) greater chance [4,9].

Chronic steroid use may predispose patients to peripheral vascular complications following percutaneous TAVR [4]. Using data from the National Inpatient Sample database, hospitalizations with transcatheter aortic valve replacement (TAVR) and surgical aortic valve replacement (SAVR) procedural codes in patients on maintenance chronic steroid therapy were identified from 2012 to 2019 [1]. This study sought to investigate the impact of prolonged systemic steroid use on patients undergoing transcatheter aortic valve replacement (TAVR) in relation to their discharge disposition. Our study searched through the National Inpatient Sample Database (NIS). We aimed to demonstrate the disposition of hospitalizations with current chronic systemic steroid use who underwent TAVR.

## Materials and methods

Data source

This retrospective study used large datasets. We analyzed hospitalizations between January 1st, 2016, and December 31st, 2019, from the Nationwide Inpatient Sample (NIS). The NIS was created and is maintained by the Agency for Healthcare Research and Quality and is the largest publicly available all-payer in-patient database in the United States. It was designed as a stratified probability sample representing all non-federal acute care hospitals nationwide. Hospitals are stratified according to ownership/control, bed size, teaching status, urban/rural location, and geographic region. A multistage 20% probability sample of all hospitals within each stratum is then collected. All discharges from these hospitals are recorded and then weighted to ensure they are nationally representative. Data from 47 statewide data organizations (46 States plus the District of Columbia) encompassing more than 97% of the US population is included in the NIS 2016-2019 sampling frame. As many as 30 discharge diagnoses for each hospitalization were recorded using the International Classification of Diseases, Tenth Revision, Clinical Modification (ICD-10) in NIS 2016, and 40 discharge diagnoses and 25 procedures were coded in the NIS 2019 database. In the NIS, diagnoses are divided into principal and secondary. A principal diagnosis was the main ICD-10 code for hospitalization. Secondary diagnoses were any ICD-10 code other than the principal diagnosis. We did not require an Institutional Review Board (IRB) approval since all patient data in NIS are de-identified and publicly available. 

Inclusion criteria 

The population of interest consisted of all in-patient hospitalizations >18 years of age with a primary diagnosis or secondary diagnosis of current chronic systemic steroid use, regardless of the steroid indication. We then selected within this population those whose records showed they had undergone TAVR. We used a diagnosis code Z7952 to identify the current chronic systemic steroid use population and 02RF3, the ICD-10 procedure code for TAVR.

Exclusion criteria

Hospitalizations less than 18 years and those without a record of undergoing TAVR were excluded.

Analysis

We used SAS 9.4 (SAS Institute Inc., Cary, USA) to perform all statistical analyses for our study. We reported continuous variables as means with standard deviation (SD) and categorical variables as frequencies with percentages. We interpreted a p-value of 0.05 or less as statistically significant.

## Results

Between 2016 and 2019, we identified 44,200 TAVR hospitalizations, and 382,497 were on current long-term steroid therapy. Of these, 934 had current chronic steroid use and underwent TAVR (STEROID) with a mean age of 78 (SD=8.4). About 50% were female, 89% were Whites, 3.7% were Blacks, 4.2% were Hispanics, and 1.3% were Asians. Disposition was ‘home,’ ‘home with home health’ (HWHH), ‘skilled nursing home’ (SNF), ‘short-term inpatient therapy’ (SIT), ‘discharged against medical advice’ (AMA), and ‘died.’ 

**Figure 1:**
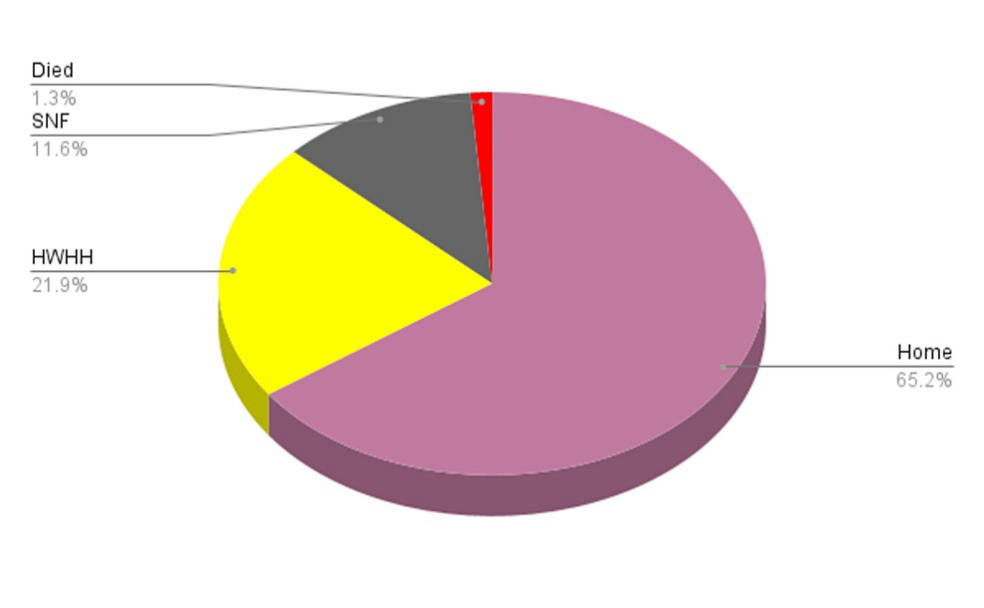
Representation of Discharge Disposition For Patients Who Underwent TAVR from 2016 to 2019 TAVR= Transcatheter Aortic Valve Replacement

A total of 602 (65.5%) were discharged home, 206 ( 22%) were discharged to HWHH, 109 (11.7%) to SNF, and 12 (1.28%) died. Only 3 and 2 patients were in the SIT and AMA groups, p=0.23. Figure 1 below is an illustration of the percentage population based on their disposition status.

The group that underwent TAVR and was not on chronic steroid therapy (NOSTEROID) had a mean age of 79 (SD=8.5), with 28731 (66.4%) being discharged home, 8399 (19.4%) to HWHH, 5319 (12.3%) to SNF, and 617 (1.43%) died p=0.17. 

**Figure 2:**
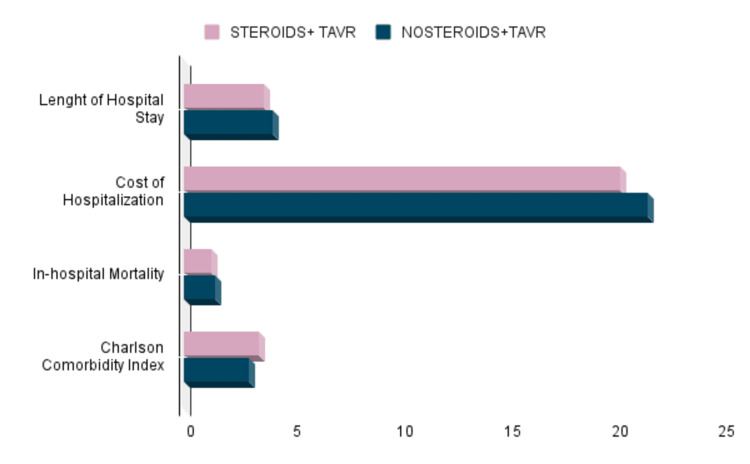
Comparing Disposition Outcomes Between Groups

Comparing the STEROID vs. NONSTEROID group, according to the CCI, the STEROID group scored higher than the NOSTEROID group; 3.5 (SD=2) vs. 3 (SD=2) p=0.0001, while for LOS, it was 3.7 days (SD=4.3) vs. 4.1 days (SD=5.3), p=0.28, and the THC was $203,213 (SD=$110,476) vs. $215,858 (SD=$138,540), p=0.15. Figure 2 below summarizes the comparison between the two groups.

## Discussion

TAVR is a widely accepted intervention for patients with aortic stenosis deemed unsuitable for conventional surgical aortic valve replacement [10]. Elderly patients benefit from this procedure mainly due to reduced complications compared to SAVR, multiple coexisting comorbidities, and their frail state. Findings from our study showed that the mean age from STEROID and NOSTEROID groups was over 78 years, consistent with other studies that revealed that TAVR is often carried out in elderly patients [11-15]. The use of chronic corticosteroid therapy is not uncommon in this set of patients due to the presence of comorbidities (e.g., rheumatoid arthritis, interstitial pneumonia, autoimmune hepatitis, polymyalgia rheumatica, systemic lupus erythematosus, multiple myositis, scleroderma) [6] which requires the use of this medication. Moreover, some of these patients are frail with multiple coexisting comorbidities, causing them to experience slower recovery and decline in functional capacity, requiring discharge other than home after the procedure [16]. 

This study showed that the discharge disposition of patients with TAVR in the STEROID group compared to that of the NOSTEROID was not statistically significant, as they had comparatively similar outcomes. Our study revealed that 66% of patients in the STEROID and NOSTEROID groups were discharged home, while just over 30% of patients from both groups were discharged home with home health (HWHH) or to a skilled nursing facility (SNF). Okoh et al. revealed that 81% of patients who had TAVR were discharged home, while 19% were discharged to locations other than home [15]. Although Okoh et al. were not specific if some of the patients benefited from HWHH, we found that over 85% of patients in both STEROID or NOSTEROID groups were discharged to home/HWHH, with patients being discharged home having a much larger percentage. Arora et al. noted that 72% of patients who had TAVR were discharged to home/home healthcare, 25% were transferred to SNF, and 1% were transferred to short-term hospital therapy [13]. Gautier et al. revealed that patients receiving systemic corticosteroids were less frequently discharged to home compared to patients not using systemic corticosteroids [16]. Both groups' high home/HWHH discharge disposition may be attributed to significant success in the TAVR procedure, low postprocedural complications, and short hospitalization (LOS). Length of stay is a critical factor responsible for in-hospital complications, such as nosocomial infections [18] and functional decline [19], which is known to be a deciding factor in the discharge disposition. It has been shown that sicker, debilitated patients are more likely to be discharged into nursing facilities [13]. 

Our study revealed that the length of hospitalization of STEROID and NOSTEROID groups was about four days, showing no significant difference between those with chronic use of steroids requiring TAVR and those without. Joshi et al. reported no significant difference in LOS in patients who had TAVR in both the STEROID and NOSTERIOD groups, as LOS for the STEROID group was 6.47 days while that of the NOSTEROID group was 5.62 days [11]. Koyama et al. reported a median LOS for the STEROID group was nine days, while that of the NOSTERIOD group was 10 days, although this difference was insignificant [5]. A separate study by Arora et al. showed a median LOS of 4 days for patients who had TAVR. This was consistent with our findings for both groups. Arora et al. noted that the LOS could be due to the use of local anesthesia and conscious sedation in TAVR and is associated with a reduction of intensive care unit days, modified techniques, small sheath sizes, and fewer postprocedural complications [13].

This study showed that the STEROID group had a mortality of 1.28% compared to that of the NOSTEROID group (1.43%), which was not statistically significant. This is consistent with that of studies done by Joshi et al. [11] and Koyama et al. [5], which revealed no differences between the two groups with respect to in-hospital mortality. However, studies have shown that chronic steroid use increases the risk of early procedural complications and mortality [11,15,17-19]. Koyama et al. reported that the STEROID group had a higher incidence of vascular complications, life-threatening bleeding, red cell transfusions, and also a significantly high rate of 1-year mortality owing to noncardiovascular death [5]. Gautier et al. also noted a significantly high rate of one-year mortality owing to noncardiovascular causes in the STEROID group [16]. Ang et al. reported a higher risk of major vascular complications, major bleeding, and aortic annulus rupture in the STEROID group compared to the NOSTEROID group [19]. Generally, patients who chronically use steroids are often complicated with multiple comorbidities and adverse effects of steroids, including tissue fragility, impaired glucose tolerance, and increased risk of infections.

Findings from our study showed the Charlson Comorbidity Index (CCI) was higher in that of the STEROID group (3.5) compared to that of the NOSTEROID group [12]. This represents significant comorbidity, taking into account the age group of these patients, severity of illness, and multiple coexisting comorbidities that are likely to be present. Unsurprisingly the CCI of the STEROID group was higher than that of the NOSTEROID group, likely due to the worsening effects of long-term use of steroids. In a study by Bouleti et al., patients who had TAVI had a mean CCI of 5.3 ± 2.3 and were seen to have relatively high in-hospital mortality and higher rates of late mortality [21]. Munoz et al. found a high CCI of 3.57 and revealed increased mortality after 30 days [21]. George et al. noted a CCI of 2.67 in patients who underwent TAVR [22] and showed no clear association between CCI and mortality [23].

We found that the total hospital charge of patients in the STEROID group was within $203,213, while that of the NOSTEROID group was around $215,858. These two groups had no significant difference in the total hospital charge. Arnold et al. analyzing the result from the PARTNER I trial, revealed that the mean cost of initial hospitalization for TAVR was $79,619 ± 40,570 [23]. McCarthy et al. reported a median cost of $50,200 for TAVR, with Medicare paying $215,770,200 nationally for TAVR in 2012 [24]. TAVR is an expensive procedure with economic implications. Considering the high-risk nature of the patient, it is performed on. It is not surprising that the cost implication is high. TAVR is reasonably cost-effective for inoperable and high-risk patients with aortic stenosis [25,26].

Strengths and limitations of the study

This study uses one of the largest hospitalization datasets of the US population. The database is useful for research, albeit designed as an administrative tool for billing purposes, and relies on the coders' accuracy, hence the possibility of overbilling, underbilling, and wrong coding. The TAVR complications were not taken into account during our study. The NIS is unable to differentiate between multiple hospitalizations from a single individual. Hence this may result in a duplication bias.

## Conclusions

The comorbidity burden of individuals on long-term steroids undergoing TAVR was slightly higher than those not on steroids undergoing TAVR. Despite this, there was no statistically significant difference in their disposition, length of stay, and hospital charges. The current practice of early hospital discharge for TAVR patients should be emphasized and encouraged.
